# Lentiform fork sign in uraemic encephalopathy: clinical image

**DOI:** 10.1093/omcr/omaf169

**Published:** 2025-09-28

**Authors:** Pramith Ruwanpathirana, Kaveendra Pathirage, Thamalee Palliyaguru

**Affiliations:** Professorial Unit in Medicine, National Hospital of Sri Lanka, Colombo 01000, Sri Lanka; Professorial Unit in Medicine, National Hospital of Sri Lanka, Colombo 01000, Sri Lanka; Professorial Unit in Medicine, National Hospital of Sri Lanka, Colombo 01000, Sri Lanka

**Keywords:** nephrology, neurology, radiology, medical imaging

Uraemic encephalopathy presents with a wide spectrum of symptoms ranging from cognitive impairment to seizures and coma, often posing diagnostic challenges. We present a case of uraemic encephalopathy with a rare but characteristic radiological sign.

A 61-year-old Sri Lankan male with end-stage kidney disease on once-weekly haemodialysis presented with three days of altered behaviour and three generalized tonic–clonic seizures. His Glasgow Coma Scale was 9/15 (E-3, V-1, M-5). He had normal pupils, diminished reflexes, and flexor plantar responses. No extrapyramidal signs or evidence of meningoencephalitis were noted. Blood pressure was 187/84 mmHg. Blood sugar, serum Na^+,^ and Ca^2+^ were normal. Serum creatinine was 13.4 mg/dl. The blood alcohol level, urinary toxicology and ketone body screenings were negative. Electro-encephalogram revealed generalized slowing. Metabolic encephalopathy, central nervous system (CNS) infection, drug/toxin overdose, autoimmune encephalitis, and CNS vasculitis were the differential diagnoses.

Non-contrast CT (Fig. 1: subset a) showed bilateral symmetrical basal ganglia hypodensities, bordered laterally by a black line corresponding to the external capsule. MRI/FLAIR revealed bilateral basal ganglia hyperintensities. In ADC map (Fig. 1: subset b) three linear hyperintensities delineated the internal and external capsules and the medullary laminae, forming the *lentiform fork sign*. A diagnosis of uraemic encephalopathy was made. Symptoms and radiological signs resolved with intensified dialysis.

**Figure 1:**
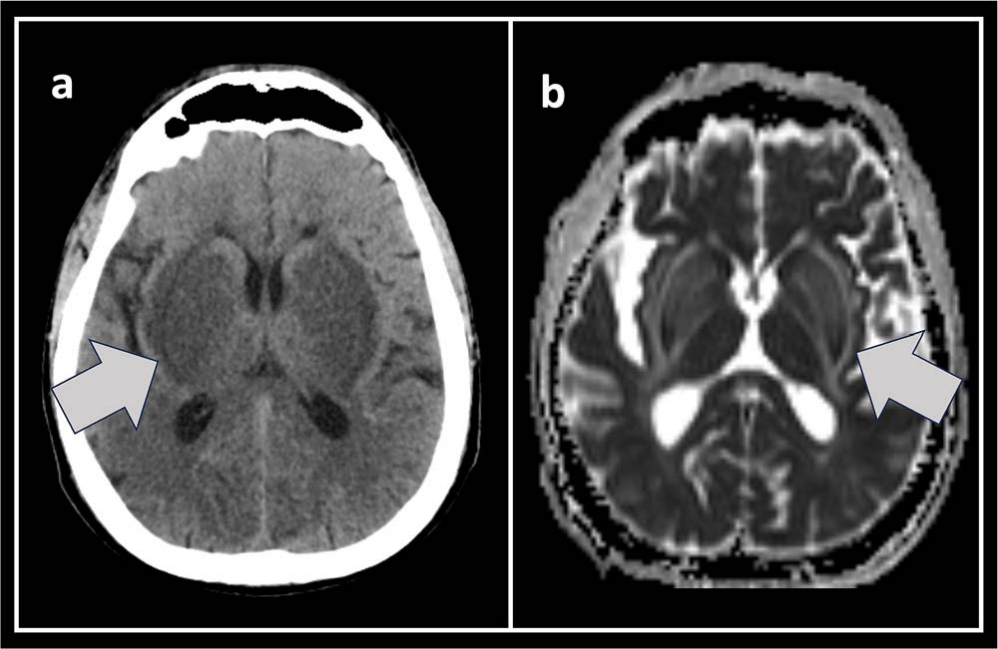
The Lentiform fork sign. The non-enhanced CT (subset a) and ADC map (subset b) with the lentiform fork sign. The arrows point to the lentiform fork. Non-enhanced CT show bilateral symmetrical basal ganglia hypodensities, bordered laterally by a black line corresponding to the external capsule. ADC map show three linear hyperintensities delineating the internal, external capsules and the medullary laminae, CT—Computed tomography, ADC—Apparent diffusion coefficient.

The lentiform fork sign represents vasogenic oedema around the basal ganglia. Oedema occur due to blood–brain barrier disruption by uraemic toxins, acidosis, and electrolyte imbalances (3). Basal ganglia are preferentially affected due to high metabolic demand and dense vasculature (3).

Incidence of lentiform fork sign was 10.3% in a study with 29 uraemic encephalopathy patients [1]. Lentiform fork sign has been described in methanol and ethanol poisoning, diabetic ketoacidosis, metformin toxicity, and dialysis disequilibrium syndrome [2].

In conclusion, the lentiform fork sign can aid the diagnosis of uraemic encephalopathy.

## Data Availability

Further clinical images will be issued upon reasonable request from the corresponding author.

## CRediT statement

PR, KP and TP were involved in patient care, imaging acquisition, manuscript preparation, and literature review.
